# Empowering medical students in the climate crisis: elective curriculum for undergraduate medical education

**DOI:** 10.3389/fpubh.2025.1679553

**Published:** 2025-10-31

**Authors:** Margaret Baldwin, Isabella Shelby, Gretchen Hoyer, Tyler Bates, Sheila Modi, Anusha Govind

**Affiliations:** ^1^School of Medicine, University of Texas Southwestern Medical Center, Dallas, TX, United States; ^2^Division of General Internal Medicine, Department of Internal Medicine, University of Texas Southwestern Medical Center, Dallas, TX, United States; ^3^Division of Infectious Diseases and Geographic Medicine, Department of Internal Medicine, University of Texas Southwestern Medical Center, Dallas, TX, United States

**Keywords:** medical education, medical student, curriculum, climate, public healthclimate health

## Abstract

**Introduction:**

Climate change has a widespread impact on human disease and population health. Despite calls for action from physician organizations, medical schools have been slow to integrate climate and health education into their curricula.

**Curriculum design:**

We designed and implemented a medical student elective focused on climate and health. The course consisted of expert-led didactics and small group discussions using case-based or journal article–based sessions to explore the intersection of climate change and health. Qualitative student feedback on the course was collected with a RedCap form after each iteration of the course.

**Results:**

Upon course completion, students reported greater comfort in understanding the impact of climate change on the health of their community and recognizing how climate change exacerbates health disparities. Students also noted that they hoped to integrate what they learned in their future medical practice.

**Discussion:**

This course demonstrates a feasible model for incorporating climate and health education into medical curricula that may be used by other medical schools. Such educational interventions may better prepare future physicians to address the health consequences of climate change and promote health equity.

## Highlights

- We developed a medical student elective course on climate change and health with the goal of increasing medical students’ fundamental knowledge of climate change and its impact on health.
- Climate change impacts health in many ways, including heat, cardiovascular, pulmonary, renal, infectious disease, nutrition, and mental health.
- At the end of the course, students reported greater understanding of the impact of climate change on health and hoped to integrate this knowledge into their clinical practice.

## 1 Introduction

Between 2030 and 2050, the World Health Organization estimates that an additional 250,000 deaths per year will be attributable to climate change (1). Climate change is an already serious contributor to poor health outcomes, including pulmonary, infectious, and heat-related diseases—and the problem only continues to grow (2). The worsening climate crisis already disproportionally affects physiologically and socially vulnerable populations, including indigenous communities, children, the older adults and people of low socioeconomic status (3, 4). Major physician organizations have endorsed the addition of climate health education to undergraduate medical education, including the American College of Physicians (ACP) (5) and American Medical Association (6). The AMA supports climate change education in medical schools so “that trainees and practicing physicians acquire a basic knowledge of the science of climate change, can describe the risks that climate change poses to human health, and counsel patients on how to protect themselves from the health risks posed by climate change… (6)”. Despite this call to action, medical schools have been slow to incorporate climate change education, with 70% of health professions schools surveyed by the Global Consortium on Climate and Health Education reporting difficulty incorporating climate health education into their existing curriculum (7–9). Despite the constant evolution of medical school curricula, many graduating medical students report that their education in climate health was inadequate (10, 11).

Physicians have a unique responsibility to understand climate health, given the importance of this topic to the health and well-being of patients and the support of large medical education organizations. Introducing climate health education early in medical training, particularly during pre-clinical years (the first one to two years of classroom-based learning in U.S. medical education prior to clinical rotations) would allow medical schools to educate future physicians early in their careers and provide an early framework for climate-informed medical care. This additionally allows educators to provide an integrated and overarching view of climate health, not just in the context of individual classes or clinical rotations. To support the next generation of physician leaders, medical schools must begin to educate their students about the impacts of climate change on the health of their patients and communities.

The concept of a climate health course incorporated into the medical school curriculum is a relatively new one. At the University of Texas Southwestern Medical Center, no formal climate health curriculum existed before the implementation of the Climate and Health enrichment elective. Through this interdisciplinary model, we sought to ensure that the information disseminated to our students was the most up-to-date and evidence-based with included opportunities for direct discussion in a student-led environment.

While other climate health curricula have been piloted at other medical schools, few data exist on the outcomes and responses of students to these changes (12). Several medical schools describe initiatives to integrate climate change and health into their curriculums on their websites, including UMass Chan, Emory, University of Minnesota, and George Washington University medical schools, though have not yet described the outcomes of these initiatives (13–16). We were able to find only a few published studies discussing the implementation and integration of climate health education into medical school curriculum (17–19). At Harvard Medical School, after introducing “Climate Change, Environment, and Health” as a core curricular theme, surveyed students and found that the majority of students agreed that climate change and health should be part of all medical student education and that the topic was valuable and important (18). Similarly, at Stanford Medical School, medical students who participated in a graduate seminar on climate change and health also agreed that physicians should be educated on climate change and that climate change should be included in the medical school curriculum (19).

While many institutions agree that this education is important, as discussed above, medical schools may struggle to implement it seamlessly into an already packed curriculum. Making changes that involve multiple courses in a medical school curriculum is a lengthy process requiring extensive review and approval by the curriculum committee. By providing this course as an enrichment elective that students were able to opt into, we were able to efficiently put together a curriculum and start providing climate health education to interested students. At the same time, we were able to use the framework, curriculum materials, and student feedback to provide to the medical school as evidence of the need for and student interest in more widespread climate health education. We hope that through this curriculum we can foster future physicians to be better climate crisis advocates, which will benefit patients across the world for years to come.

## 2 Curriculum design

We designed an elective that was provided as a 12-week non-credit enrichment elective every spring for interested students as a pilot project at the University of Texas Southwestern Medical School. The target demographic for this elective was pre-clinical students. Approximately 7–10 students were enrolled in the class during each session over the last 4 years. The small class size was intentional, as our curriculum utilizes small group discussions and projects which benefit from more intimate discussion of the material. As this elective was created as an introductory course with integrated lessons on the basic science of climate change, there was no prerequisite knowledge required. We worked with the Texas Physicians for Social Responsibility organization and course coordinators at other medical schools to identify local and regional experts in the field of climate health to give lectures specifically tailored for our course and students. The course included a faculty sponsor and medical student coordinators to facilitate the required tasks for this elective.

The groundwork for this course began with polling faculty at our institution and educators at other institutions who are interested in climate health. We then compiled a list of topics that addressed the effects of climate change on general medical practice as well as other specialty care, including infectious diseases and pulmonology. One lecture in our course specifically focused on the local microclimate in North Texas, while other lectures focused on the national or global perspective of climate and health. It was important to include a multidisciplinary lens to our curriculum including nutrition and mental health topics to provide a holistic view of the impacts of climate change on health. To give our students tangible skills to impact patient care, we included lectures on climate science, advocacy, and how to take a climate informed history. To address existing health inequities in certain patient populations, we included lectures on the disproportionate effects of climate change on the health of marginalized communities. Finally, information on the impact of the healthcare industry on climate change was included to serve as an avenue for potential change-making.

A sample syllabus and a complete list of topics included in the most recent iteration of the course is included in Appendix I. The overall goal in creating this curriculum was to equip students with skills to address climate health and fill this knowledge gap in the core medical student curriculum. The course was delivered in hybrid format with some sessions in person and some virtual to accommodate speakers. The sessions were held during lunch hours. Small group sessions led by student liaisons were also included in the class format and were comprised of journal clubs, video discussions, and project presentation days to allow for increased student interaction and engagement with course material. The grading of the course was pass/fail and based on attendance and completion of a project. The guidelines for the student projects are included in Appendix 2.

All students received a RedCap survey at the end of the course to provide qualitative feedback on the effectiveness of the course in meeting the stated learning objectives as well as to elicit suggestions for improvement. The domains we aimed to evaluate with the survey included course quality and effectiveness, learner experience and general feedback, and applications to clinical practice. The students received the following questions to evaluate these aspects of the course:

- What were examples of excellence in this elective?
- What are opportunities for improvement in this elective.
- What are further topics you would like to see discussed in this course and do you have any other general feedback?
- How will you incorporate knowledge from this course into your future patient care and history taking?
- Do you think this is an important topic for all medical students?

We reviewed the responses to the surveys after each year of the course, allowing us to make changes and improvements year to year in response to student comments. For the purposes of this analysis, the study team grouped similar responses into clusters. From these grouped responses, representative themes were generated to describe patterns across student feedback received.

## 3 Results: course evaluation and feedback

During our review and analysis of student feedback, several themes emerged, including course content and relevance, teaching methods, structure and engagement, utility in clinical practice, further integration into the medical school curriculum, and empowerment and responsibility. These themes are further explored below with representative quotes collected from the surveys included.

### 3.1 Course content and relevance

Overall, students appreciated the variety of topics included in the curriculum and found the course to be comprehensive and interesting. Students appreciated that the course began with an introduction to the science behind climate change, allowing everyone to have a baseline understanding before learning about specific effects on health. Students also enjoyed learning directly from physicians about the different ways climate change impacts human health, some of which were new to the students.

#### 3.1.1 Student quotes

- “Learning from physicians about health impacts from the climate was extremely helpful and made the focus of the course very clear.”
- “I really enjoyed how comprehensive this class was as I feel it touched on a lot of different aspects of the impact of climate change, many of which I didn’t know existed.”
- “I thought it was super interesting to learn about all of the different topics discussed in the class.”
- “I loved the variety of speakers that cohesively made for a very broad course.”
- “This elective was a detailed and organized course about climate change and its impact on health. There was a great variety of speakers and activities that really added to our education.”
- “I really enjoyed all of the lectures. I appreciated that the class started with a lecture about the science behind climate change so that we were all on the same page. I learned a lot of things I hadn’t really associated with climate change such as the adverse effects of climate change and mental health.”

### 3.2 Teaching methods, structure, and engagement

Students appreciated the combination of lectures and discussion-based sessions, highlighting that in-person sessions allowed for more engagement and open discussion. Though the virtual sessions were convenient and ideal for lectures, several students also recommended having more in-person discussion sessions. Students also felt the length of sessions and organization of the elective was appropriate and that lunchtime sessions fit well into the pre-clinical schedule.

#### 3.2.1 Student quotes

- “Loved the class. I loved the discussion sessions that were in person.”
- “The lecturers all presented fantastic information with clinical and scientific reasoning.”
- “I really enjoyed the journal and ted talk discussions, so I think if we could incorporate more discussion time that could be really nice!”
- “I would love to have more discussion-based sessions like the one we had with the videos.”
- “Zoom is convenient, but in person lectures + food will result in more engagement.”
- “More purely conversation days; allow students to pick topics and come discuss them early in the course.”
- “I really enjoyed the discussion sessions and the presentation from the Dallas community organization, and it would be great to have more similar sessions in the future.”

### 3.3 Utility in clinical practice

Students were able to clearly see the clinical relevance of the topics discussed and how climate directly affects patients, highlighting heat exposure as something to especially consider for the patient population in the Dallas area. Some students mentioned that they had not considered the importance of a patient’s environment on their health and appreciated the opportunity to learn how to take an environmental history. Several students mentioned that they would take what they learned from this course into their future practice, remembering to consider environmental or climate factors when seeing patients in the future. Some also suggested including even more practical examples of how to include climate awareness and education in clinical practice.

#### 3.3.1 Student quotes

- “I think so much of this curriculum, especially heat and air pollution, affect nearly all patient populations.”
- “I really enjoyed hearing about concrete ways to incorporate advocacy and mindfulness about climate change into clinical practice.”
- Students also highlighted the importance of learning about taking an environmental history:
- “It was also helpful for us to learn how to take an environmental history, as I had never considered that being an aspect of care before.”
- “I’ll keep in mind the topics we discussed and incorporate them into my regular history taking when possible.”
- “I will certainly have higher suspicious for heat-related illness in our local community and consider how a patient’s environment is a significant social determinant of health.”
- “I hope to be more mindful now when I take a social history to discuss the environmental status of one’s household. Now that I have much more knowledge about pollution and the socioeconomic inequalities regarding poor sanitation, I hope that I can discuss these topics more openly with patients and brainstorm little things they can do to ensure a healthier living space.”
- “I really enjoyed the lectures that addressed the clinical significance of climate change. I would like to see more practical examples of how to include climate change education in a clinical setting.”

### 3.4 Further integration into the medical school curriculum

After completing the course, students felt that this was an important topic for all medical students, saying that it should be integrated into the general curriculum. Several students gave ideas for where it could be added to the curriculum including the integrated medicine blocks as many topics align with the organ systems already covered. Other students suggested including the information in Strive and Colleges, both of which are longitudinal courses for all medical students at UT Southwestern which are aimed at further developing professional identity and clinical skills.

#### 3.4.1 Student quotes

- “I think there were many components of this class, especially related to high heat’s effect on health, that should and could be incorporated into our general medical curriculum.”
- “I absolutely think these are important topics for all medical students because it affects each of us and our future patients. I think just as every IM block has an embryology, physiology, pathology, statistics, microbio, pathology, and pharmacology section, each block can also afford to have a 15-min lecture on climate change related to that organ system. Climate considerations can also be incorporated into our colleges time (while seeing patients or during the social determinants of health colleges session).”
- “I think it is a topic that can be weaved into the IM blocks curriculum or perhaps in Strive. Even starting with one mandatory talk about climate change and health students and can you see this being more integrated in that is interactive can go a long way.”
- “I would definitely advocate for this to be in the general curriculum. I think it would be great to have to apply this into our colleges sessions.”
- “I definitely think that climate-related changes to health outcomes, disease prevalence, geographical disease prevalence, targeted treatment should definitely be integrated into the standard medical student curriculum.”

### 3.5 Empowerment and responsibility

Several students discussed that as future physicians they had a responsibility to understand climate change and that future physicians and other healthcare professionals must be aware of environmental impacts on patients both at an individual patient level and within the broader healthcare system. For some students, the curriculum also fostered a new awareness of their potential role in addressing climate change, highlighting the unique position physicians hold in society from which to conduct advocacy work.

#### 3.5.1 Student quotes

- “Climate change is not a scientist-only topic; it is a part of every human’s life, and the more that it can be incorporated into workforce productivity, the better. As physicians, our job is to educate people about healthier lifestyles, and that includes combating climate change. Even simple things like discussing water conservation and reducing carbon emissions can go a long way, especially because we have the privilege to often be seen as leaders in the community.”
- “Physicians should be educated on the resources available in their local communities to assist patients and understand the impacts climate change is having locally. I think physicians can also serve a broader role in getting involved with organizations aimed at improving sustainability in healthcare and advocating for climate justice at local and national levels.”
- “Physicians have a responsibility to help local advocacy groups and using our voices to reinforce what they are saying!”
- “I think this is a really important topic and after taking the elective it was clear that climate change will have huge impacts on patients and healthcare in the future. It’s essential that medical students and anyone working in the healthcare is aware of how climate change is impacting human health.”

## 4 Discussion

Overall, this course was successful in introducing the topic of climate change and health to medical students and demonstrating clinical relevance, as evidenced by the themes generated from qualitative student feedback. Students found the content covered in the course to be interesting, organized, and clinically relevant. They appreciated the opportunity to learn about these topics directly from physicians. In terms of course structure and teaching methods, students appreciated the mix of lectures and discussion sessions, enjoying the chance for conversation about these topics with their peers. Students felt that this topic was important for all medical students and supported the integration of climate change and health into the broader curriculum. Finally, students described feeling a sense of responsibility as physicians to continue the conversation about climate change in their future practice and potentially getting involved in climate advocacy on a broader scale.

As future clinicians, we anticipate that the students who have completed this course will have increased awareness of the environmental factors that may affect their patients’ and community’s health and be better able to counsel patients on environmental risks, as evidenced by student responses in the feedback collected in each iteration of the course. These findings align with those of the other medical schools that have begun to introduce conversations about climate change and health into the curriculum (17–19). This suggests that in addition to the discussion among major physician organizations about this topic, that medical students are also interested and motivated to learn more about how climate change is affecting their future patients and practice, and to learn about strategies to address these issues and opportunities to get involved in advocacy (5, 6).

The sense of responsibility and interest in climate change and health instilled in the students by the course is further evidenced by one student’s final project. One student’s project led to UT Southwestern Medical School’s approval of incorporating climate change and health education longitudinally throughout the entire medical school’s pre-clinical curriculum, which has the potential to be a very impactful outcome from this course. The framework and curriculum materials developed for this elective course as well as the student feedback collected were instrumental in highlighting the importance of integrating climate health education into the core curriculum to the curriculum committee. Topics such as temperature susceptibility among patients taking certain medications, the impact of temperature on medication pharmacodynamics, and the changing epidemiology of certain bacterial, fungal, and viral illnesses have been added to the new medical school curriculum as a result for all incoming medical students. This example highlights how when equipped with knowledge and understanding of how climate change is impacting human health can be empowered to find ways to address the impacts of climate change and human health. Physicians, as trusted leaders within many communities, have a unique position from which to advocate for climate justice and advance planetary health (20, 21).

Reviewing survey results after each iteration of the course allowed us to make improvements in response to student feedback. For example, during the second year of this course, the curriculum from the previous year was expanded to include speakers from a local community organization to provide students with examples of local environmental and climate-related health risks, as well as information about local advocacy efforts. During the third and fourth year of the course, we began holding many sessions in person in response to these comments to allow for more participation in the lecture-based didactics in addition to the small group discussion sessions.

We feel that this curriculum is readily adaptable for use at other institutions, and we hope this example will be valuable to other educators interested in incorporating climate change and health in medical education. A faculty sponsor and two medical student liaisons were sufficient to complete planning and administrative tasks related to the course: recruiting faculty to prepare lectures, advertising the course, setting up Zoom lectures, sending announcements to students and taking attendance. The medical student liaisons gained valuable leadership and organizational skills in preparing for and facilitating the sessions. We recommend using the sample syllabus and course schedule with the key topics we covered included in Appendix I as a framework for structuring a similar elective course. Many medical schools have the opportunity for students to design and initiate elective courses with the help of a faculty sponsor. Alternatively, the organizers could use this framework to create a proposal to be presented to the medical school curriculum committee for integration within the core curriculum.

While much of the didactic information presented in the course is widely applicable, some of the topics were specifically relevant to the North Texas area. For future iterations of the course, course organizers should update discussion session topics and specific examples used throughout the didactic lectures to the relevant geographic region and needs of local patient populations.

Integrating climate health education into core medical school curricula is essential; however, starting with an elective structure may be a more feasible and efficient way to begin disseminating the information and generating interest in the topic among students and faculty. Additionally, starting with a smaller group of students allowed us to quickly adapt the course and make changes based on student feedback that were useful in presenting the topic to the medical school curriculum committee as important to integrate longitudinally within the core curriculum. It is recommended that this course be used similarly by other medical institutions to start elective courses or develop materials to update their respective core medical school curricula. By establishing this course as proof-of-concept, we hope that it is useful in preparing for the incorporation of information related to climate change and health into the broader pre-clinical curriculum.

### 4.1 Limitations

We had small groups of students, likely limiting the breadth of perspectives represented in the course evaluation results. Course participation was also voluntary; thus, many students likely had a preexisting interest in addressing and understanding climate change prior to the course. Finally, our evaluation form captured student feedback right at the end of the course and provides no measurement of long-term outcomes of the course. Further research is needed in this area to evaluate long-term outcomes of climate education for medical students and assessing student interest and response to the topic on a wider scale.

## 5 Conclusion

This course allowed students to develop a better understanding of the impact of climate change on the health of patients and communities, both now and in the future. The changing climate is already impacting patient health around the world, and it is imperative that future physicians are educated on this subject. This course empowers medical students to be physician advocates for both their patients and the planet. The next steps of this project include sharing the structure of this curriculum for easy implementation at other medical and healthcare professional schools. We have also created a longitudinal curriculum to integrate climate health into the pre-clinical curriculum at UT Southwestern Medical School.

## Data Availability

The original contributions presented in the study are included in the article/Supplementary material, further inquiries can be directed to the corresponding author.
