# Supplementary material for: Empowering medical students in the climate crisis: elective curriculum for undergraduate medical education
Source: Front Public Health. 2025 Oct 31;13:1679553. doi: 10.3389/fpubh.2025.1679553 (PMC12616737; doi:10.3389/fpubh.2025.1679553)
Supplement: Supplementary file 1 [file Data_Sheet_1.pdf]

Appendix I: Sample Syllabus and List of Curriculum Topics

Course title: Climate and Health - Spring 2025

Objectives:

1) Students will learn the science behind climate change and understand the basic tenets of natural and human causes that worsen global warming

2) Students will be able to identify adverse effects of climate change in their patients' health in various aspects from infections, pulmonary health, and nutrition

3) Students will learn the exaggeration of existing healthcare disparities in populations affected adversely by climate change

4) Students will learn how to appropriately take a history on a patient while addressing environmental exposures and concerns that could be contributing to their concerns

5) Students will be able to list concrete changes that they can implement in their own lives to help decrease their individual carbon footprint

6) Students will learn the tools and skills to advocate in the local, state, and national level to impact healthcare practices affected by climate change

Format:
Didactics and small group discussion. Sessions will be a hybrid of in-person and virtual. All sessions will be on Tuesdays from 12-12:50 pm.

Student Evaluation:

Grades will be pass/fail. Attendance is required to receive credit for the course.

Sample Schedule:

**Week**	**Topic**
1	Introduction to the course/project
2	Heat and adverse effects on health
3	Heat and health: a prescription for change
4	Climate change effects on pulmonary health
5	Nutrition and climate
6	Climate change impacts on mental health and migration
7	Taking an environmental history
8	Journal club/video discussion
9	Standardized patient interview
10	Disparate impacts of climate change on marginalized communities, the local Dallas-Fort Worth "microenvironment"
11	Book club
12	Effects of the healthcare industry on climate
13	Student Project Presentations
14	How to be an informed and effective advocate

Appendix II: Student Project Guidelines

Students may choose from the following ideas for projects listed below or propose an alternate project to be completed and presented during the last class session. Projects may be done individually or in small groups of 2-3 students.

1) Write a 3-paragraph essay:

   -A reflection piece of what your baseline knowledge was about this topic, what you have learned during the course, and what you will be taking forward in your clinical practice.

   -A more in-depth discussion focusing on one of the topics covered.

2) Patient education: Develop educational material (flyer/brochure) geared towards patients to mitigate environmental impacts on health.

3) Student education: Develop educational material geared towards students (could be for elementary, secondary, college, or medical students) regarding topics involving the impact of climate change on health.

4) Get involved in an advocacy activity and then discuss your involvement with the class
